# Feasibility Study for a Chemical Process Particle Size Characterization System for Explosive Environments Using Low Laser Power

**DOI:** 10.3390/mi11100911

**Published:** 2020-09-30

**Authors:** Jesse Ross-Jones, Tobias Teumer, Susann Wunsch, Lukas Petri, Hermann Nirschl, Mathias J. Krause, Frank-Jürgen Methner, Matthias Rädle

**Affiliations:** 1 Center for Mass Spectrometry and Optical Spectroscopy, Mannheim University of Applied Sciences, Paul-Wittsack-Straße 10, 68163 Mannheim, Germany; susann.wunsch.sw@gmail.com (S.W.); lukas.petri@stud.hs-mannheim.de (L.P.); m.raedle@hs-mannheim.de (M.R.); 2 Lattice Boltzmann Research Group, Institute for Mechanical Process Engineering and Mechanics, Karlsruher Institut für Technologie, Straße am Forum 8, 76131 Karlsruhe, Germany; jesse.ross-jones@partner.kit.edu (J.R.-J.); hermann.nirschl@kit.edu (H.N.); mathias.krause@kit.edu (M.J.K.); 3 Chair of Brewing Science, Department of Food Technology and Food Chemistry, Technische Universität Berlin, Seestraße 13, 13353 Berlin, Germany; frank-juergen.methner@tu-berlin.de

**Keywords:** angular dependence, side-scattering, low laser power, particle size measurement, recursion model

## Abstract

The industrial particle sensor market lacks simple, easy to use, low cost yet robust, safe and fast response solutions. Towards development of such a sensor, for in-line use in micro channels under continuous flow conditions, this work introduces static light scattering (SLS) determination of particle diameter using a laser with an emission power of less than 5 µW together with sensitive detectors with detection times of 1 ms. The measurements for the feasibility studies are made in an angular range between 20° and 160° in 2° increments. We focus on the range between 300 and 1000 nm, for applications in the production of paints, colors, pigments and crystallites. Due to the fast response time, reaction characteristics in microchannel designs for precipitation and crystallization processes can be studied. A novel method for particle diameter characterization is developed using the positions of maxima and minima and slope distribution. The novel algorithm to classify particle diameter is especially developed to be independent of dispersed phase concentration or concentration fluctuations like product flares or signal instability. Measurement signals are post processed and particle diameters are validated against Mie light scattering simulations. The design of a low cost instrument for industrial use is proposed.

## 1. Introduction

In many industrial processes, such as production of colors, paints or abrasives, filtration, emulsification, crystallization, precipitation as well as aerosol generation, monodisperse particles are monitored [1,2,3,4,5]. Some of these processes, such as color or paint production, as well as aerosol generation, are known for their explosion risk due the use of solvents or dust formation (explosive class 20 or 21) [6,7,8,9]. In industrial production environments, where explosive concentrations of volatile organics or dust occur, most instruments are not permissible due to the danger of ignitions and explosions [9,10]. Many of the production environments grouped into this classification [6,11], allow only laser class 1 [12], where laser power less than 1 mW can be used. Established particle size measuring instruments on the market do not employ such low power detection systems and instead focus on reliable high quality off-line measurements of unknown products [13,14,15,16]. Comparable measuring systems, which also measure light scattering over a range of angles, work with laser powers in the 5 mW range [17]. By applying prior knowledge to the measurement process, such as refractive index, chemical composition or particle form, unknown characteristics (mean size, concentration, and local and/or temporal distribution) of the particles can be determined using simplified instrument arrangements. The unique features of this work are the possibility of on-line measurement of particle size with very low laser power (5 µW) in combination with very fine measurement step size (2°) in an angular range between 20° and 160° together with the presented mathematical prediction model.

Furthermore, this measurement technology offers advantages for applications in the chemical and pharmaceutical industries, as it uses low light input, reducing the probability that product alterations occur during process measurement. In food engineering, for example, undesirable processes, such as contamination from bacteria [18,19] and haze formation through proteins and polyphenols [20,21], lead to turbidity of otherwise clear liquids and decreased product quality and shelf live. Early detection of such impurities, using on-line or in-line systems, is therefore desired. Of particular advantage are measuring designs which continuously provide results with in-line monitoring in micro channels, which are optimized to save product, energy and experimental time during recapture development and product optimization. One such example would be the in-line monitoring of silk fibroin during self-assembly, whereby tunable production of 200 to 1500 nm protein microspheres, in the presence of ethanol, is performed [22].

This work focuses on the use of static light scattering for particle size measurements. A wide range of particle sizes can be measured using light scattering. In a typical experimental setup, electromagnetic radiation of a precise wavelength is directed onto a sample and the scattered wave is analyzed with a suitable detector [13]. The momentum and the energy difference between the scattered light and the incident light are used to characterize the structure and dynamics of the particle being measured. Since scattering techniques are non-destructive, they are well suited for in-line studies or on-line studies after a dilution process [13].

There are four predominant methods using light scattering measurements: static light scattering (SLS), dynamic light scattering (DLS), turbidimetry/nephelometry and diffractometry [13]. In this work, the static light scattering measurement method is applied, which has been used for several years in material analysis to determine the particle diameter or its shape [23,24,25]. With this measurement method, particles within a range of diameters can be examined, from the sub-micrometer range up to the millimeter range [23,26]. Caumont-Prim et al. for example use 3 selected angles [24], and Li et al. use 6 angle in the 30–130° range [27], whereby measurements over 71 different angles in an angular range between 20° and 160° are performed here. Various proposals have been presented to perform particle size recognition using recursion algorithms, with multi angle measurement data and a simulation database based on Mie simulations [24,27,28]. Additionally, UV VIS spectroscopy can be used to estimate the particle size in situ [29,30]. Van Eerdenbrugh et al. for example, measure particle size using UV-VIS for particles between 300 and 400nm [30], whereas in this work particle sizes are measured in the range between 300 nm and 1000 nm.

This work presents a measurement methodology which enables the estimation of particle size using an automated light scattering measurement system. A novel way to mathematically preprocess the measured static light scattering signal is presented, for an approximation of particle size using Mie theory. The developed method, based partially on MSE (mean square error) minimization, compares positive and negative slope intervals of the scattered intensity between measurement and simulation data to determine the particle diameter. The algorithm compares data of measured and simulated light scattering based on the shape of the signal, and is independent of absolute intensity values, finding matches between both sets of data despite large variations in magnitude not achievable with least squares’ based methods.

This proposed method allows different particle systems (validated here with silica and polystyrene) to be characterized in-line, and has been tested over a range of diameters (from 300 to 1000 nm). Customized photon multipliers (CPMs) are used together with extremely low laser power (under 5 µW). In this work polystyrene and silica particles are used for the feasibility study due to their availability in a monodisperse form. In order to estimate the particle size, the measured scattering patterns are evaluated against Mie simulations conducted using MiePlot [31]. In order for the particle size to be estimated, the physical behavior of light is employed, whereby light refracts when encountering solid matter. Depending on the particle size and the difference between the refractive index of the particle and that of the surrounding medium, the diffraction or scattering pattern is significantly different and characterizable. In the past, light, neutron and X-ray scattering methods have been extensively cited in other works where the structure and dynamics of particles and macromolecules in multicomponent systems are investigated [13,25,32,33,34,35,36,37,38,39,40,41,42].

The objective of this work is to demonstrate the measurement of particle light scattering over a continuous range of angles with a low power laser, thereby overcoming limitations resulting from current particle size detection methodologies.

## 2. Materials and Methods

For on-line analytical particle characterization, a model is required for the scattering of light by a sphere of a given radius and refractive index [33,43,44]. A solution to this problem has existed for some time [44], however, it has only received practical use with the development of powerful computation capacity [45]. The modelling simplification, known as Mie theory, is a mathematical model that characterizes the scattering of light by particles when the particle size and wavelength of light have similar order of magnitude [33,44,46]. Though it is now known as Mie theory, Debye [47] and Lorentz [43] also deduced comparable solutions at the same time. For this reason, the names Mie–Debye theory, Mie–Lorentz theory, Debye theory or Lorentz theory, appear today when researching aerosols, suspensions or emulsions [25,48,49,50].

Mie scattering is based on a mathematical characterization of the electromagnetic scattering of a plane wave when encountering a sphere. The incident plane wave and the scattered electromagnetic field are described as a series of spherical wave functions. In this work, the simulation program MiePlot is used [31], which is based on the works of Bohren and Huffmann [45]. The light scattering depends on the incident light wave, the refractive indices of the scattering medium and of the surrounding system, the scattering angle and the particle size. In the light scattering simulations, all quantities are kept constant with exception of the particle size. These simulations are compiled to create a database which serves as the basis for the particle size prediction method. For the surrounding medium, water is used with a temperature of 20 °C. Each particle type has a corresponding refractive index. A wavelength dependent refractive index being used since the refractive index varies with the wavelength of the incident light. The refractive index values were taken from a database for silica and polystyrene [51] and are summarized in Table 1.

**Figure 1:**
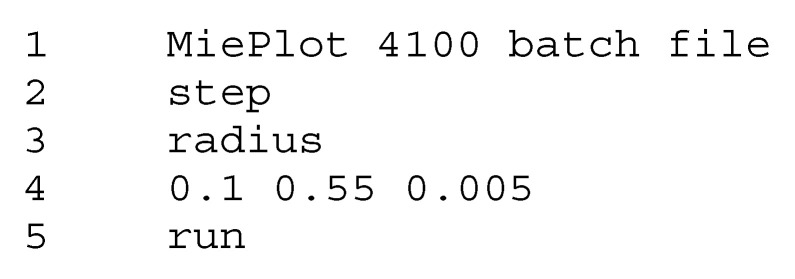
Batch file for MiePlot to automate stepping through particle sizes for intensity versus scattering angle simulations.

With MiePlot, the intensity versus scattering angel is simulated for a 532 nm light source, from 0° to 180° for particles diameters between 200 and 1100 nm in 10 nm step sizes. For each angle a moving average of ±2.5° is formed in order to compensate for the angle of reception of the lens. Once the relevant simulation parameters have been entered including the refractive indices for the particle and medium, the process of stepping through the required particle diameters is accomplished through the use of a batch file as shown in Figure 1. MiePlot then saves the requested signals to a single file.

### 2.1. Approximation and Curve Fitting

The size of the measured particles is determined by measuring the angle dependent intensity and comparing theory and experiment. The measurement signals are processed and thereafter evaluated in order to estimate particle size, which is predicted by the best fit. First the signal is approximated using sinusoidal functions [52]. This approximation aids in smoothing out the measurement signal and identifying signal features which can be used to estimate the particle size. Following the approximation, our proposed method is used to find the closest matching measurement signal from a database of light scattering simulations.

Different models can be used to approximate measurement data depending on the type of signal. In order to increase the quality of fit, a model is first selected that best describes the signal, such as a linear, polynomial or sinusoidal models [53,54]. These models can be expressed by generalized functions. The aim of the approximation is to determine the coefficients so that the deviation from the measurement data points is minimal. A frequently used method is the least squares method. First, a system of Equations of the form Ax = B is created for this purpose, see Equation (1), in which the matrix A of the data points x_1_ to x_n_ is multiplied by the coefficient matrix and is equated with the associated matrix B of the Y-values.

(1) $$\left(\begin{array}{cc} 1 & x_{1}\\ 1 & x_{2}\\ \begin{array}{c} \ldots \\ 1 \end{array} & \begin{array}{c} \ldots \\ x_{n} \end{array} \end{array}\right)\times \left(\begin{array}{c} a_{1}\\ b_{1} \end{array}\right)=\;\left(\begin{array}{c} y_{1}\\ y_{2}\\ \begin{array}{c} \ldots \\ y_{n} \end{array} \end{array}\right)$$

In the next step matrix A is transposed. The transposed matrix A^T^ is multiplied by matrix A and matrix B to matrix C and D, respectively as shown in Equation (2).

(2) $$A^{T}\times A=\left(\begin{array}{ccc} 1 & 1 & \begin{array}{cc} \ldots & 1 \end{array}\\ x_{1} & x_{2} & \begin{array}{cc} \ldots & x_{n} \end{array} \end{array}\right)\times \left(\begin{array}{cc} 1 & x_{1}\\ 1 & x_{2}\\ \begin{array}{c} \ldots \\ 1 \end{array} & \begin{array}{c} \ldots \\ x_{n} \end{array} \end{array}\right)=\left(\begin{array}{cc} n & x_{1}+x_{2}+\ldots +x_{n}\\ x_{1}+x_{2}+\ldots +x_{n} & x_{1}^{2}+x_{2}^{2}+\ldots +x_{n}^{2} \end{array}\right)=C\;A^{T}\times B=\left(\begin{array}{ccc} 1 & 1 & \begin{array}{cc} \ldots & 1 \end{array}\\ x_{1} & x_{2} & \begin{array}{cc} \ldots & x_{n} \end{array} \end{array}\right)\times \left(\begin{array}{c} y_{1}\\ y_{2}\\ \begin{array}{c} \ldots \\ y_{n} \end{array} \end{array}\right)=\left(\begin{array}{c} y_{1}+y_{2}+\ldots +y_{n}\\ x_{1}\times y_{1}+x_{2}\times y_{2}+\ldots +x_{n}\times y_{n} \end{array}\right)=D$$

The calculated matrices C and D thus convert the system of Equations into Equation (3).

(3) $$C\times \left(\begin{array}{c} a_{1}\\ b_{1} \end{array}\right)=D$$

This system of Equations is solved using the Gaussian–Jordan algorithm or Cramer’s rule, whereby the fitting coefficients a1 and b1 can be determined. To quantify the quality of the fitting, the mean square error mean squared error (MSE) can be calculated (see Equation (4)). The MSE is composed of the sum of the squared differences of the measuring points y_i_ and the corresponding approximation points $\hat{y}$ averaged over the number of measuring points n.

(4) $$MSE=\;\frac{1}{n}\overset{n}{\underset{i=1}{\sum }}{\left(y_{i}-\hat{y}\right)}^{2}$$

The smaller the MSE, the greater the quality of fit. In order to compensate for measurement noise, the measurement data points are approximated using a functionalized model. The functionalized models used in this case are a series of sine Equations as shown in the following Equations (5) and (6).

(5) $$f\left(x\right)=\;\;a_{1}\times \sin\left(b_{1}\times x+c_{1}\right)+const\;\;\;\;\;\;\;\;\;\;\;\;\;\;\;$$

(6) $$\;\;\;\;f\left(x\right)=\;\;a_{1}\times \sin\left(b_{1}\times x+c_{1}\right)+a_{2}\times \sin\left(b_{2}\times x+c_{2}\right)+const$$

**Figure 2:**
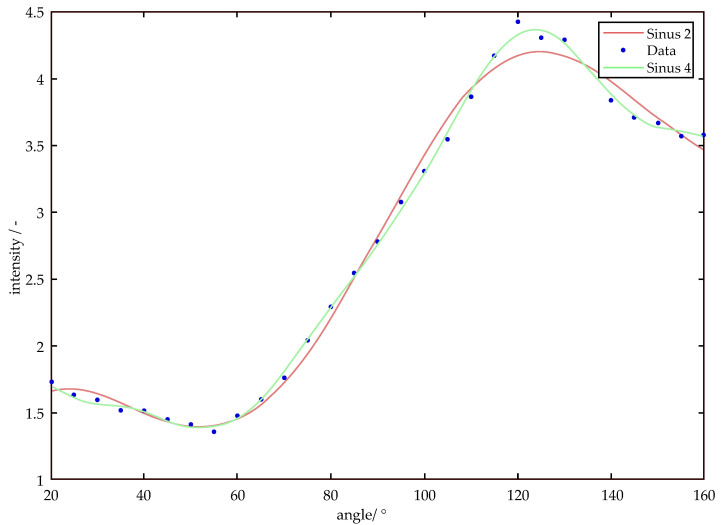
Approximation of measurement data by the sum of 2 and 4 sinusoidal terms.

The appropriate function is selected by determining the minimum MSE. Typically, curves with zero or one extremum can be modeled with a single sine (Equation (5)), whereas three or more extremes require multiple sine functions (Equation (6)), which is the case for particles smaller than 650 nm. In order to approximate larger particles and to improve the approximation of smaller particles, a total of 5 sinusoidal functions are used, an example is shown in Figure 2. However, especially with small particle sizes, care must be taken that the measurement curves are not overdetermined, since small measurement outliers and fluctuations may be included in the approximation.

Different methods can be used to compare curves in order to qualitatively determine the curve with the highest agreement from a set of curves [27,28,55]. Here the derivatives of the curves will be analyzed. The proposed method implements curve shape matching following a similar method as Buchin et al. [56], where the curve is divided into intervals and positive and negative slopes are compared.

In the first step of the comparison, the curves are partitioned and divided into two areas, one of which includes the intervals I_p_ to I_q_, in which the slope of the two curves have the same sign, while the other area is composed of the remaining intervals I_r_ to I_s_ where the slopes have opposite sign. For each of the two areas, each area is integrated over the respective intervals, see Equation (7). The variables represent a measure for the congruence of the two curve.

(7) $$a_{same\;sign}=\;a_{ss}=\overset{I_{q}}{\underset{I_{p}}{\int }}\frac{1}{\left|\;\frac{\partial y_{1}}{\partial x}-\frac{\partial y_{2}}{\partial x}\;\right|}$$

$$a_{oposite\;sign}=\;a_{os}=\;\overset{I_{s}}{\underset{I_{r}}{\int }}\left|\;\frac{\partial y_{1}}{\partial x}-\frac{\partial y_{2}}{\partial x}\;\right|$$

By forming the reciprocal, the variable a_ss_ increases as the difference between derivatives decreases, i.e., higher agreement of the curves, whereby the positive effect is amplified. By computing Equation (7) on all curves to be compared, two vectors containing the positive and negative congruence of each curve is obtained. In the next step, two vectors are divided element wise to determine the overall congruence, as shown in Equation (8). Where f is described in Equation (10). The variable a_sum_ has the same dimensions as a_ss_ and a_os_.

(8) $$a_{sum}=\;\frac{a_{ss,norm}\times f_{ss}}{a_{os,norm}\times f_{os}}$$

Equation (9) shows Equation (8) in vector notation, where n corresponds to the number of curves to be compared.

(9) (asum,1…asum,n)=  (ass,norm,1…ass,norm,n)×(fss,1…fss,n)(aos,norm,1…aos,norm,n)×(fos,1…fos,n) 

To be able to compare the values a_ss_ and a_os_, the values within the vector are normalized to their respective minimum and maximum. The minimum is not normalized however to zero, so that the curve for which a_os,norm_ is minimal does not result in division by 0. To further enhance the prediction factors f_ss_ and f_os_ are used, as shown in (8). These factors are formed from the number of intervals with the same and opposite sign, N_ss_ and N_os_ respectively, as shown in (10). The factor z describes a number of power 10, where z is strictly greater than 10.

(10) $$f_{ss}=\;\frac{N_{ss}}{N_{ss}+N_{os}}\times z$$

**Figure 3:**
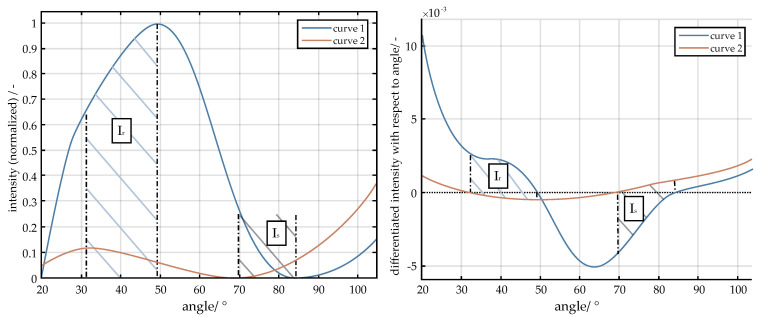
Classification of curves and division into intervals based on their gradient direction ((**a**) data normalized; (**b**) data normalized and differentiated)**.**

To determine the curve with the highest match, the maximum is simply found from the vector a_sum_. An example of curve comparison is shown in Figure 3.

**Figure 4:**
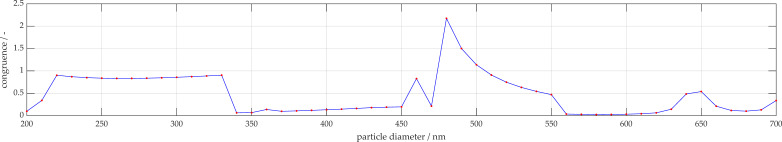
Example of a congruence diagram of the 500 nm scattering pattern compared to the simulation.

Figure 4 provides a congruence diagram, allowing visualization of the agreement between the fitted measurement curves and the simulation curves. The highest peak corresponds to the predicted particle size.

**Figure 5:**
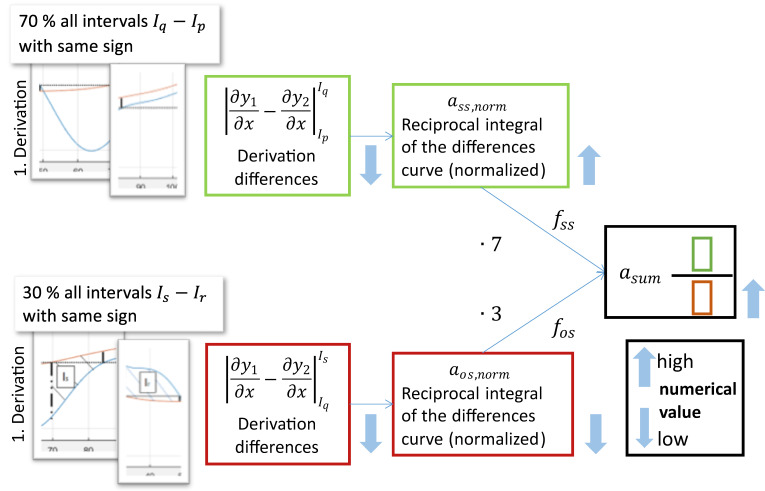
Diagram illustrating the calculation of congruence to determine the particle size of a fitted curve from the simulation database.

In Figure 5 the logic for determining the congruence between the measurement and simulation curves is illustrated. High congruence corresponds to equidirectional slopes in both the measurement and simulation curves.

### 2.2. Particle Systems

Two particle systems are used in the feasibility studies. Spherical Micromer^®^ polystyrene particles (PS), purchased from Micromod Partikeltechnologie GmbH (Rostock, Germany), with various particle diameters ranging from 0.5 µm (PDI < 0.2, PDI = polydispersity index) to 2 µm (PDI < 0.2). Spherical sicastar^®^ silicate particles, sourced from Micromod Partikeltechnologie GmbH in Germany, with particle diameters ranging from 0.5 µm (PDI < 0.2) to 1 µm (PDI < 0.2).

### 2.3. Experimental Setup

The following laboratory setup is used to demonstrate the particle characterization using a low power laser. The design of the industrial instrument will differ from this design to reduce the number of moving components and is described in the results section.

**Figure 6:**
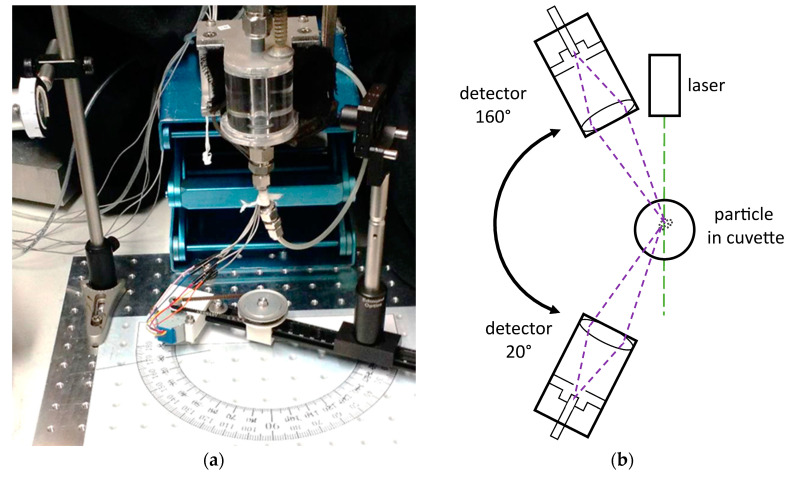
(**a**) Adjustment of cuvette; (**b**) adjustment of detector.

For the measurements, a laboratory setup was constructed to measure the light scattered at angles ranging between 20° and 160°, as shown in Figure 6. While measurement of only a few angles may be sufficient for characterizing small particles in the Rayleigh range, measurements of larger particles in the Mie range are often hindered by ambiguity due to aliasing of the increased number of peaks present. To overcome this ambiguity, the measurements are made with a detector which can be adjusted in 2° increments over a range from 20° to 160° around the measurement cuvette, as shown in Figure 6. The increased measurement resolution allows for particles scattering light throughout the Mie regime to be characterized. A linear 532 nm laser module (CW532-005L, Roithner Lasertechnik, GmbH, Vienna, Austria) with a nominal power of 5 mW is used. The laser was mounted at a 45°angle to the scattering field to capture both, parallel and perpendicular polarization. Various color filter foils from LEE Filters Worldwide, USA are used for attenuation. The selected filter combinations are 2x 024 Scarlet for the green laser with a final power of 4.1 µW [57]. The CPM CM 93YE provided by ProxiVision, are used as detectors. The advantages of using these photon counters are the low background noise produced of only three to ten counts per second, together with the maximum sampling rate of 15 megacounts per second. This allows for measurements of high dynamic range (5 levels of magnitude), which are simply not possible using comparatively noisy analog amplifiers. The CPMs are powered by a 5 V power supply and provide the input signal to a quTools Time-to-Digital Converter, which is connected to a PC via a USB interface. The measuring environment consists of a round glass cuvette with an inner diameter of 1 cm, which is embedded in an aluminum casing. The overlap between the focus of the laser and the detection area is below 1 mm^3^, thus easily adaptable to micro channels. The holder of the glass cuvette is mounted on a rail which is attached to a laboratory lifting platform. Thus, the glass cuvette has two degrees of freedom, displacement horizontally on the rail and vertically by adjusting the laboratory lifting platform. The laser is mounted on a vertical rod, independent of the test table.

The laser can also be fitted with different filters for further attenuation. The sample liquid is pumped from a storage container into the measuring chamber by means of a peristaltic pump. The sample is introduced into the measuring chamber from below to avoid standing air bubbles. Furthermore, the sample is constantly stirred to maintain homogeneity. The detector of the test apparatus is mounted on a rotating axis driven by a stepper motor. The center of the detector lens is located below the laser beam. The lens has a diameter of 12.5 mm and a focal length of 10 mm (FRP0510, THORLABS, Newton, NJ, USA). The lens collects the scattered light which is then focused on a glass fiber which is connected to the CPM. The distance between the lens and the center of the cuvette is 50 mm. The pivot point is located vertically below the center of gravity of the measuring cuvette. The laser beam hits the measuring solution in the center of the glass cuvette and perpendicular to the volume flow direction. In addition to measuring in a low light environment, the measuring apparatus (laser, measuring cuvette, and detector) is completely enclosed during the measuring process to reduce extraneous light influences on the measurements.

## 3. Results

A CPM on a rotational axis was used to measure light scattering over a continuous range of angles. Polystyrene particles of size 300 and 1000 nm, as well as silica particles of sizes 300 (310) nm, 500 (507) nm, 700 (681) nm, 900 (900) nm and 1000 (987) nm were measured (exact manufacturer particle sizes are given in parentheses). Dispersions of 0.015 g/L of particle to distilled water were prepared for the measurements. The results of the silica particle analysis are presented here, followed by the results of the polystyrene particles.

**Figure 7:**
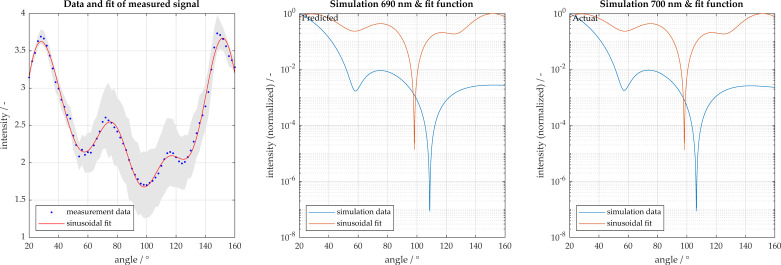
Particle size characterization for 681 nm silica particles (particle size displayed is rounded to 680). Light scatter measured in 2° increments from 20° to 160°. (**a**) Measurement and sinusoidal fit with 95% confidence intervals shown in gray; (**b**) comparison to best fit result; (**c**) comparison to actual particle size.

**Figure 8:**
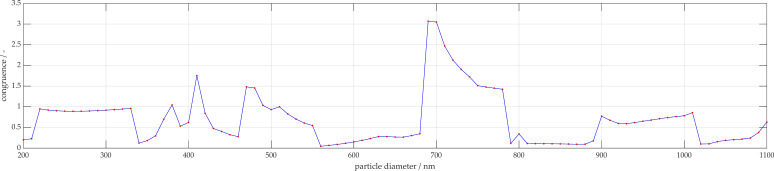
Overall congruence of 681 nm measurement to simulation database.

Detailed analysis of the silica particles of diameter 681 nm are shown in Figure 7 and Figure 8. The particles were characterized accurately with a size of 690 nm. Figure 6a contains a representation of the measurement signal with the sinusoidal fit function, which was described previously. Additionally, 95% confidence intervals are displayed, whereby the sample size was 65 measurements. Figure 7b displays the simulation curve found to be most congruent with the measurement signal. Figure 7c displays the expected simulation curve, known a priori. Figure 8 displays the vector a_sum_, described previously, which is the congruence of the measurement signal to each simulation, where a single peak is present at approximately 700 nm.

The results presented in Table 2 are an overview of the characterization analysis of the measured silica dispersions. The measured 310, 507, 681, 900 and 987 nm silica particles are accurately characterized to within 6.5%.

**Figure 9:**
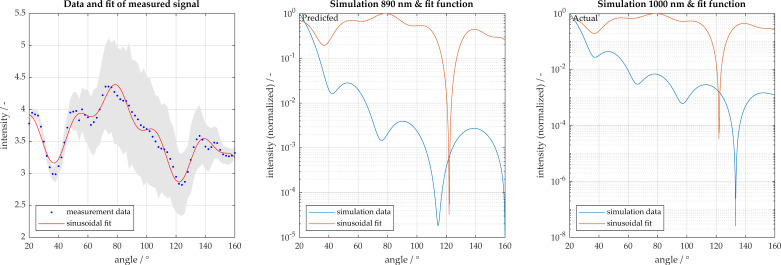
Particle size characterization for 1000 nm polystyrene particles. Light scatter measured in 2° increments from 20° to 160°. (**a**) Measurement and sinusoidal fit; (**b**) comparison to best fit result with 95% confidence intervals shown in gray; (**c**) comparison to actual particle size.

**Figure 10:**
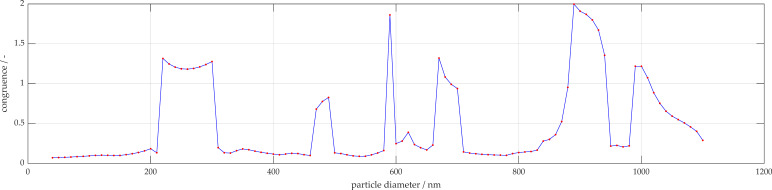
Overall congruence of 1000 nm measurement to simulation database.

**Figure 11:**
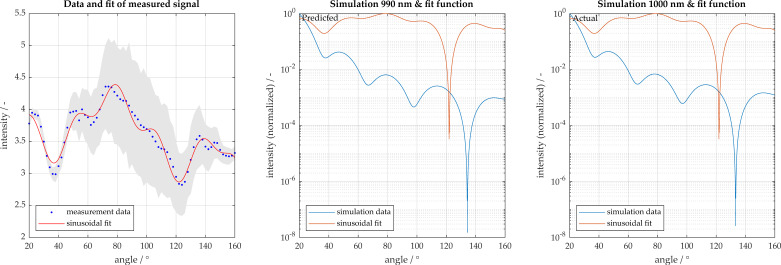
Particle size characterization for 1000 nm polystyrene particles after filtering simulation database for signals with similar number of local extrema. Light scatter measured in 2° increments from 20° to 160°. (**a**) Measurement and sinusoidal fit; (**b**) comparison to best fit result with 95% confidence intervals shown in gray; (**c**) comparison to actual particle size. Using the signal feature of number of local extrema allows confounding signals to be filtered out and prediction is improved.

**Figure 12:**
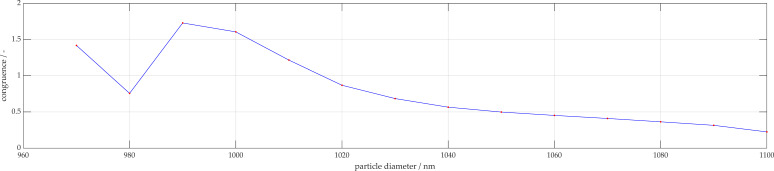
Overall congruence of 1000 nm measurement to simulation database after filtering for signals with similar number of local extrema.

Polystyrene particles of sizes 500 and 1000 nm were also analyzed. The 500 nm particles are accurately characterized as being 460 nm. Figure 9a displays a representation of the measurement signal for 1000 nm with the sinusoidal fit function. A 95% confidence interval is also displayed, whereby the sample size here was 55 measurements. When reviewing the analysis of the 1000 nm particles, these are incorrectly characterized as 890 nm as shown in Figure 9b. In order to improve the characterization, the number of local maxima in the measured signal are considered, and only the simulations with similar number of extrema are used for characterization. These results are shown in Figure 10. When using only the filtered potential particle sizes, the algorithm is able to narrow down potential fits, more accurately estimating 990 nm. The corresponding simulation is shown in Figure 11 with the associated congruence in Figure 12. This demonstrates the importance of measuring with high angular resolution in order to be able to resolve the individual peaks in the scattering signals.

The results presented in Table 3 are an overview of the characterization analysis of the measured polystyrene dispersions. The measured 500 and 1000 nm polystyrene particles are accurately characterized to within 8%.

## 4. Discussion and Future Work

Light scattering measurements of 310, 507, 681, 900 and 987 nm silica particles and 500 and 1000 nm polystyrene particles were performed and analyzed. The particle size prediction results demonstrate that the silica particle sizes were characterized to within 6.5% and the polystyrene particles to within 8%. These results may be further improved by measuring and simulating with a second wavelength to decrease the number of potential particle size matches.

In particular, these results show the importance of using a high angular resolution when measuring light scattering over a broad range of angles, otherwise aliasing of peaks occurs and false deductions are made when interpreting the particle diameter, as was the case with the measurement of 1000 nm polystyrene particles. Since 71 measurements between 20° and 160° with 2° angle increments were taken, the number of peaks resolved in the signal could be leveraged to filter out unlikely particle size candidates.

While this work obtains the mean particle size, a second iteration is planned whereby the particle size distribution is estimated. The workflow entails first characterizing the mean particle size as described here, followed by a second characterization whereby the measured signal is compared to simulated Mie scattering signals with fixed mean particle size and increasing diameter distribution width. In this way, the mean particle size as well as particle distribution are procedurally characterized.

**Figure 13:**
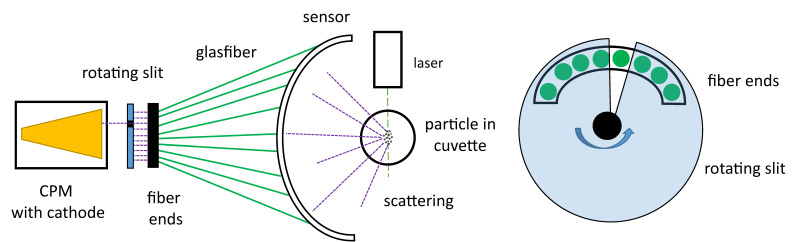
(**a**) Fiber sensor in a semi circle form to detect scattered light over a range of angles and forward it to the customized photon multiplier (CPM). Between the sensor and the CPM is a rotating slit which allows only the signal from one fiber at a time to reach the CPM. (**b**) The fiber ends are placed next to each other in an arc, and through each rotation of the slit the array of fibers is exposed sequentially.

Based on these results an approach for an industrial optical setup is proposed as seen in Figure 13. The goniometer is replaced by a fiber bundle in order to remove the need to rotate a single optical fiber around the measurement cuvette. The fiber ends are lined up next to each other in an arc, whereby 71 fibers are needed. A rotating slit allows only one optical fiber to be exposed at a time and forward photons to the cathode of the CPM. By adjusting the sampling rate of the CPM’s and the rotational speed of the slit, a measured value can be recorded for each angle. With a slot rotation rate of 20 Hz and a sampling rate of 20 kHz of the CPM’s and 71 fibers, several values per second per fiber can be recorded facilitating measurement of changing particle sizes.

An important consideration for the design of the industrial sensor is the small diameter of the fibers and the amount of collected light. The laboratory setup used a 12.5 mm diameter lens, while the optical fibers have a diameter of 1 mm. This results in an effective area-reduction of 156.25. To partially compensate, the fiber ends will be placed at 20 mm from the measurement cuvette instead of 50 mm. A minimum distance of 20 mm is required to ensure that all fibers can be placed next to each other. The resulting scattered light intensity therefore increases by a factor of 6.25. The resulting light intensity is therefore: 6.25/156.25 = 1/25 in the proposed design. The design continues to be feasible, since a factor of 200 still remains before the currently used 5 µW exceeds the 1 mW limit.

## 5. Conclusions

In this work we demonstrate that for an in-line cuvette based particle sizer, a laser power of only 5 µW and an integration time of 1 ms were sufficient for particle size measurement. By scaling the power maximum up to the allowed 1 mW, a sampling rate of 20 kHz can be achieved. By overcoming the need for high powered lasers for particle sizing, measurements in micro channels as well in explosive environments and a combination of both become possible.

This was accomplished using a photon counting module able to measure photon scattering over 5 levels of magnitude. Side scattering was measured in an angular range between 20° and 160° around the cuvette, whereby the optical probe of the detector in the test apparatus was rotated automatically around the cuvette. The measurement took place in 2° and 5° incremental steps. In the proposed industrial design, this setup will be replaced by a fixed angle arrangement and a rotating slit.

An algorithm is proposed and used to estimate particle diameter from scattering light measurements, over a continuous range of angles, in the Mie regime. Since the methodology is only dependent on the position of maxima and minima as well as slope, the evaluation is not dependent on absolute intensity, and is unaffected by factors such as changes in concentration. Good prediction accuracy was demonstrated for two particle systems, silica particles between 300 and 1000 nm and polystyrene particles of 500 and 1000 nm. The importance of measuring over a continuous range of angles was also demonstrated, reducing signal aliasing effects occurring when only few angles are measured. Based on the presented results, a potential setup was introduced to implement a similar measurement system in industrial environments, where explosion safety is necessary.

## Figures and Tables

**Table 1:** Wavelength dependent refractive index for silica and polystyrene [51].

Wavelength (µm)	Silica	Polystyrene
0.43584	1.4667	1.6170
0.47998	1.4635	1.6070
0.58756	1.4585	1.5916
0.70652	1.4551	1.5825

**Table 2:** Overview of silica size characterization results.

Polarization	Predicted Result (nm)	Data Sheet (nm)	Relative Error
Unpolarized	330	310	6.5%
Unpolarized	480	507	5.3%
Unpolarized	690	681	1.3%
Unpolarized	880	900	2.2%
Unpolarized	960	987	2.8%

**Table 3:** Overview of polystyrene size characterization results.

Polarization	Predicted Result (nm)	Data Sheet (nm)	Relative Error
Unpolarized	460	500	8.0%
Unpolarized	990	1000	1.0%
